# Electroacupuncture reduces blood glucose by regulating intestinal flora in type 2 diabetic mice

**DOI:** 10.1111/1753-0407.13323

**Published:** 2022-10-04

**Authors:** Jing An, Lingli Wang, Shuangning Song, Lugao Tian, Qingqing Liu, Minhui Mei, Wenhua Li, Shi Liu

**Affiliations:** ^1^ Department of Gastroenterology, Union Hospital, Tongji Medical College Huazhong University of Science and Technology Wuhan China

**Keywords:** electroacupuncture, gut microbiota, ICC, inflammation, type 2 diabetes, 肠道菌群, 2型糖尿病, 电针, 炎症, ICC

## Abstract

**Background:**

The development of diabetes is closely related to the gut microbiota in recent studies, which can be influenced by intestinal motility. A few studies report that electroacupuncture (EA) can lower blood glucose. EA can promote colonic motility and influence gut microbes. In this study, we explored the effect of the EA on blood glucose level in mice with type 2 diabetes (T2D) and its mechanism.

**Methods:**

The T2D mice model, fecal microbiota transplantation mice model, and Kit^W/Wv^ mice model （Point mutation of mouse W locus encoding kit gene）were used to investigate the effect of EA on blood glucose as well as the mechanism; The blood glucose and insulin resistance level and the intestinal flora were evaluated. The level of intestinal junction protein, inflammatory cytokines in the serum, interstitial cells of Cajal content, and colonic motility were detected. Lastly, the IKKβ/NF‐κB‐JNK‐IRS‐1‐AKT pathway was explored.

**Results:**

EA lowered the blood glucose level, altered the gut microbiota, and promoted colonic motility in T2D mice. EA‐altered microbiota decreased the blood glucose level and insulin resistance in the antibiotics‐treated diabetic mice. EA increased tight junction protein, lowered inflammatory factors, and regulated the IKKβ/NF‐κB‐JNK‐IRS‐1‐AKT pathway in the liver and muscles. EA could not reduce the blood glucose and regulated gut microbiota in the Kit^W/Wv^ mice model.

**Conclusions:**

EA promoted intestinal motility to regulate the intestinal flora, thereby reducing the level of systemic inflammation, and ultimately lowering the blood glucose by the IKKβ/NF‐κB‐JNK‐IRS‐1‐AKT signal pathway.

## Graphical abstract

**Highlights**

- Electroacupuncture (EA) regulates the intestinal flora by promoting intestinal motility.
- EA regulated the intestinal flora to reduce the level of systemic inflammation.
- EA lowering the blood glucose by the IKKβ/NF‐κB‐JNK‐IRS‐1‐AKT signal pathway.

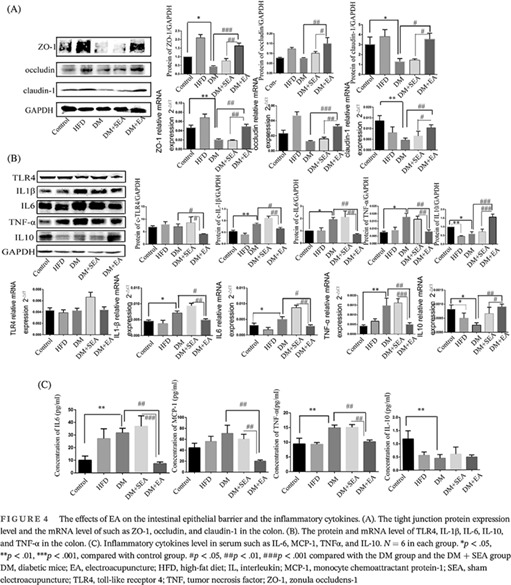

## 摘要

**背景:**

最近的研究表明, 糖尿病的发展与肠道菌群密切相关, 肠道菌群可能会受到肠道运动的影响。一些文献报道电针(EA)可以降低血糖。 EA可以促进结肠运动并影响肠道微生物。在这项研究中, 我们探讨了 EA 对 2 型糖尿病 (T2D) 小鼠血糖水平的影响及其机制。

**方法:**

采用T2D小鼠模型、FMT(粪菌移植)小鼠模型和Kit^W/Wv^小鼠模型, 研究电针对血糖的影响及其作用机制, 评估血糖和胰岛素抵抗水平以及肠道菌群。检测肠连接蛋白水平、血清和结肠炎性细胞因子水平、ICC(Cajal间质细胞)含量及结肠运动性。最后, 探索了 IKKβ/NF‐κB‐JNK‐IRS‐1‐AKT 通路。

**结果:**

1. EA降低了T2D小鼠的血糖水平, 改变了肠道菌群, 促进了结肠运动。 2. EA 改变的微生物群降低了 ABX(抗生素)治疗的糖尿病小鼠的血糖水平和胰岛素抵抗。 3. EA增加紧密连接蛋白, 降低炎症因子, 并调节肝脏和肌肉中的IKKβ/NF‐κB‐JNK‐IRS‐1‐AKT通路。 4. EA不能降低Kit^W/Wv^小鼠模型中的血糖和调节肠道菌群。

**结论:**

电针通过IKKβ/NF‐κBJNK‐IRS‐1‐AKT信号通路促进肠道运动, 调节肠道菌群, 从而降低全身炎症水平, 最终降低血糖。

## 1 INTRODUCTION

The incidence rate of type 2 diabetes (T2D) has increased in recent years. Medicines clinically have some side effects although with hypoglycemic effect. Electroacupuncture (EA) with its safety and good reproducibility could reduce blood glucose level and improve insulin resistance in patients and animals with T2D,^1^, ^2^ suggesting it may be an effective alternative treatment for T2D. Moreover, recent studies have found that development of T2D is closely related to the intestinal flora. An intestinal flora imbalance, such as lower abundance, changed the composition of the flora in patients with T2D.^3^ Individuals with low abundance of intestinal flora were more likely to exhibit insulin resistance, and increased richness of intestinal flora could improve glycometabolism.^4^ In addition, a few studies have confirmed EA can restore the flora diversity of mice with insulin resistance.^5^, ^6^

Intestinal flora imbalance seriously destroyed the intestinal barrier and increased intestinal permeability, causing intestinal inflammation and inflammatory factors thereby entering circulation, resulting in a systematic low‐grade inflammatory state.^7^ Some reports demonstrated the destroyed intestinal barrier and higher inflammation in mice with insulin resistance.^8^ Inflammatory factors in circulation reduced insulin sensitivity by regulating the IKKβ/NF‐κB‐JNK‐IRS (insulin receptor substrate 1)‐AKT pathway in liver and muscle tissues.^9^, ^10^ The effects of EA on intestinal inflammation have been verified,^11^ and our recent study reported the protective effect of EA on the intestinal barrier in mice with colitis.^12^ Thus, whether EA can maintain the integrity of the intestinal barrier by regulating the intestinal flora, thereby reducing inflammation and promoting the normal conduction of insulin signaling through IKKβ/NF‐κB‐JNK‐IRS‐1‐AKT pathway in liver and muscle tissues of diabetic mice, needs to be further investigated.

It is worth considering how EA regulates the intestinal flora. It has been found that changes in intestinal motility can affect the dynamic balance of intestinal flora. Polyethylene glycol‐fed mice with short intestinal transit time have increased beneficial bacteria and decreased destructive bacteria.^13^ Interstitial cells of Cajal (ICC) are the initiators and coordinators of intestinal smooth muscle movement. The survival, development, and proliferation of ICC deeply rely on the activation of its membrane receptor KIT (c‐kit) by the unique ligand, membrane‐bound stem cell factor (mSCF). Our previous study showed that EA can maintain ICC, thereby accelerating delayed colonic transmission in diabetic rats.^14^ Therefore, we used the ICC‐deficient mice to investigate whether colonic motility dependent on ICC is a medium for the effect of EA on intestinal flora.

Taken together, the purpose of this study was to investigate whether EA can promote colonic motility by maintaining ICC, thereby regulating gut microbiota, and ultimately decreasing blood glucose level in mice with T2D, as well as its mechanism.

## 2 METHODS

### 2.1 Animals

Male C57BL/6 mice and male Kit^W/Wv^ mice (23 g ±1 g, provided by the Jackson Laboratory) were used in accordance with the Animal Care Guidelines of Tongji medical college. Mice had free access to sterile water and standard diet for 2 weeks for acclimatization in the Specific Pathogen Free‐level environment.

### 2.2 Type 2 diabetic mice model

The diabetic mice model was established using a high‐fat diet (containing 60% fat, 20% protein, and 20% carbohydrate) for 4 weeks followed by an intraperitoneal injection of low‐dose streptozotocin (STZ, 100 mg/kg, dissolved in 0.05 M sodium citrate buffer, pH 4.5). The normal control mice were given normal chow (containing 15.8% fat, 20.3% protein, and 63.9% carbohydrate) and received the intraperitoneal injection of equal vehicle citrate buffer. Fasting blood glucose (FBG) was measured 1 week after the injection, and mice with glucose level > 11.1 mmol/L were considered as T2D.

### 2.3 The groups and EA treatment

**FIGURE 1:**
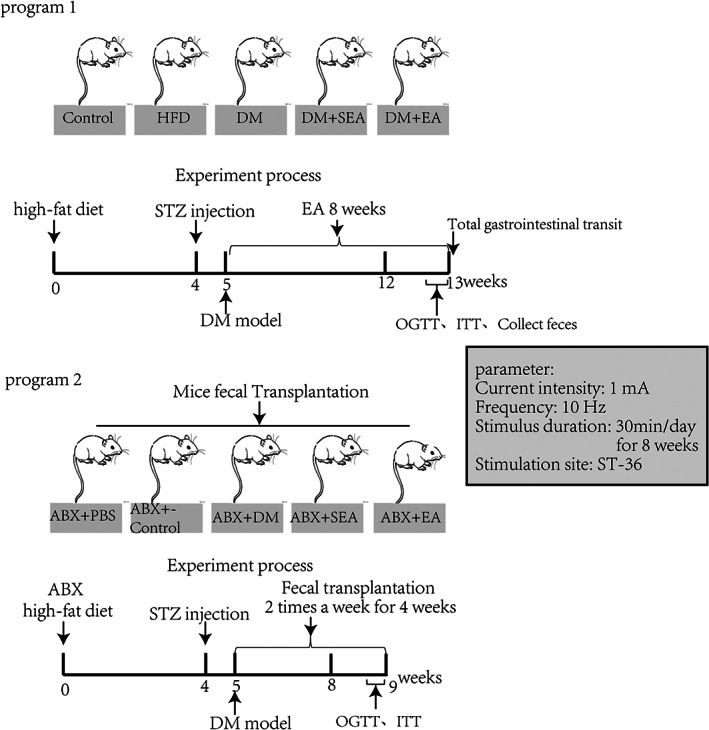
Groups and experimental procedure. Program 1. Diabetic mice and EA treatment process. Program 2. ABX‐treated diabetic mice and FMT process. ABX, antibiotics; EA, electroacupuncture; FMT, fecal microbiota transplantation; HFD, high‐fat diet; ITT, insulin tolerance test; OGTT, oral glucose tolerance test; SEA, sham electroacupuncture; STZ, streptozotocin

Fifty mice were randomly divided into five groups: normal control group (Control, without EA treatment), High‐fat diet group (without EA treatment), diabetic group (DM, without EA treatment), diabetic with sham EA group (DM + SEA, acupuncture but no electric current, 30 min/day), and diabetic with EA group (DM + EA, 10HZ, 1‐3 mA, 30 min per day). EA was executed at 8 a.m. every morning for 8 weeks since building the successful DM model. The electrical stimulator (G6805‐2A; Shanghai Huayi Medical Instrument Co. Ltd., Shanghai, China) was used in our study. The mouse ST‐36 was at the posterolateral knee (2 mm under the fibular head) of the bilateral hind limbs. Steel needles (0.16 × 7 mm) were inserted into the acupoint at a depth of 2‐3 mm. To eliminate the restraint stress, mice were fastened in a cage 30 min/day for a week before EA treatment, and the EA treatment lasted 8 weeks. The groups and EA treatment are shown in Figure 1, program 1.

### 2.4 Random blood glucose and fasting blood glucose detection, oral glucose tolerance test, and insulin tolerance test

Random blood glucose (RBG) and FBG of mice were monitored once every 2 weeks. On the last 2 days of the last week of EA treatment, oral glucose tolerance test (OGTT) and insulin tolerance test (ITT) were performed. Mice fasted overnight for 12 h were gavaged with 2 g/Kg glucose. Blood glucose level was detected before glucose administration (at 0 min), and at 30, 60, and 120 min after glucose intake.

Similarly, the day after the OGTT, mice fasted for 5 h and then received an injection of 0.75 U/Kg insulin. The mice blood glucose level was detected at 0, 15, 30, 60, 120, and 180 min after the injection. The total area under the curves (AUC glucose) of OGTT and ITT was calculated using the trapezoidal rule.

### 2.5 Total gastrointestinal transit and fecal parameter measurements

On the day after the end of 8 weeks of EA treatment, after an overnight fast, the mice were given a semiliquid solution (0.2 ml) containing 5% Evans blue (E2129, Sigma) and 1.5% methyl cellulose (M0262, Sigma) by gavage. Then the fecal pellets were monitored and the time for the expulsion of the first blue pellet was determined. Mice were kept in the metabolizable cage for 24 h with no restraint of food and water. The number of pellets per cage was recorded and weighed (wet weight). Pellets were then dried overnight at 65°C and reweighed until reaching a constant weight (dry weight).

$$\text{Pellet frequency}:\frac{24\;\text{hours total pellets number}}{24\;\text{hours}}\;\times 100\%$$

$$\text{Fecal water content}:\frac{\left(\mathrm{wet}\;\text{weight}-\mathrm{dry}\;\text{weight}\right)}{\mathrm{wet}\;\text{weight}}\times 100\%$$

### 2.6 Detection and analysis of intestinal microbes

In the last day of EA treatment, the mouse feces were collected by the stimulated defecation method. Mice were immobilized, their tails were lifted, and the lower abdomen of the mice was gently pressed with a sterile cotton swab. After defecation, the fresh feces were collected in a coded sterile EP tube using a sterile pinch. The feces were dispensed 3–4 capsules per tube, stored at −80°C, and transported on dry ice. The feces were thawed during the assay, and the V3 and V4 variable regions of the prokaryotic 16S rDNA in the sample were amplified by specific primers to construct a high‐throughput sequencing library and the 16S rDNA variable region sequence was analyzed on the Illumina MiSeq sequencing platform. The composition and abundance of prokaryotic microorganisms in the intestine of mice were identified.

### 2.7 Mice sacrifice and samples collection

After 8 weeks of EA, all the mice were euthanized by cervical dislocation. The mice colon tissues, liver tissues, and skeletal muscle (the quadriceps muscle samples) were obtained and stored in −80°C. Blood samples were collected and then centrifugated (3000 rpm, 10 min), and the supernatants were frozen at −80°C.

### 2.8 Blood serum analysis

The serum insulin was analyzed by ELISA, and the serum glycosylated hemoglobin (HbA1c) was quantified using a commercial kit (Nanjing Jiancheng Biology Engineering Institute). Tumor necrosis factor alpha (TNF‐α), interferon‐γ, interleukin (IL)‐6, and IL‐10 level in serum were analyzed by CBA assay (BD Biosciences, New Jersey, USA). Briefly, the standard protein samples were multiple proportion diluted, and six kinds of microparticles were prepared and mixed with the sample serum. Phycoerythrin‐conjugated antibodies were added to the mixtures, incubating for 3 h at room temperature away from light. A BD LSR Fortessa X‐20 system was applied to assess individual cytokine concentrations based on the fluorescence intensities.

### 2.9 Immunohistochemical staining

Whole fresh colon tissues were put into the cold Krebs solution containing 118.1 mmol/L NaCl, 4.8 mmol/L KCl, 25 mmol/L NaHCO3, 1.0 mmol/L NaH2PO4, 11.1 mmol/L glucose, 1.2 mmol/L MgSO4, 2.5 mmol/L CaCl2, 95% O2, and 5% CO2, and then the contents were washed away. The mucosa and submucosa of the colon tissues were removed, and the muscular layer was fixed in the acetone solution for 10 min. Normal donkey serum with 0.3% Triton X100 was used for overnight at 4°C. The samples then were incubated with primary antibody and secondary antibodies. Prepared colon specimens were examined using confocal microscope (Olympus, Tokyo, Japan).

### 2.10 Western blotting

Frozen tissues (50 mg of colon tissues [a random segment of colon], 30 mg of liver tissues, and 50 mg of muscle tissues) were respectively homogenized in cold radioimmunoprecipitation assay buffer containing phosphatase inhibitors (Protease Inhibitor Cocktail Tablets, Roche, 04693159001), and the ratio of protein to lysate is 100:1 (mg/ml). The concentration of extracted protein was detected by bicinchoninic acid protein assay. Total protein (100ug) was resolved on sodium dodecyl sulfate polyacrylamide gel electrophoresis and transferred onto a nitrocellulose membrane. Membranes were incubated with first antibody and GAPDH, followed by incubation with corresponding horseradish peroxidase‐linked secondary antibodies. The bands were visualized by an enhanced chemiluminescence agent (ThermoFisher, USA). Quantity One software (Bio‐Rad Technical Service Department, Version 4.6.2) was used to perform the band densitometry analysis. The reagents information as shown in Table 1.

**TABLE 1:** Reagents information

Reagents information
Antibody
Rat anti‐**c‐kit** antibody, eBioscience, San Diego, CA, 1:100
Rabbit anti‐**Ano1** antibody, Abcam, Cambridge, MA, USA), 1:200
Dylight 488 with goat anti‐rat IgG, Abbkine, California, USA, 1:150
Dylight 594 with goat anti‐rabbit IgG, Abbkine, California, USA, 1:150
Goat anti‐**c‐Kit** polyclonal antibody, R&D Systems, Minneapolis, USA1000; 1:1000
Goat anti‐**SCF** antibody; R&D Systems, Minneapolis, USA, 1:1000
Anti‐CD284/**TLR4** antibody, Arigo biolaboratories, ARG54702, 1:250
**IL‐1β** Mouse (3A6) mAb, Cell Signaling Technology(CST), #12242s, 1:500
**IL‐6** polyclonal Antibody, ABclonal Technology, A0286, 1:500
Rat anti‐**IL‐10** antibody, R&D Systems, Minneapolis, USA, 1:500
Mouse anti‐**TNF‐α** antibody, Santa cruz Biotechnology, sc‐52 746, 1:250
Rabbit anti‐**Zo‐1** polyclonal antibody, ThermoFisher scientific, TG273738, 1:500
Mouse anti‐**occludin** antibody, Santa cruz Biotechnology, sc‐133 256, 1:200
Rabbit anti‐**claudin‐1** polyclonal Antibody, ThermoFisher scientific, TE268283, 1:250
**IKK**α/β(phospho Ser176/177) polyclonal antibody, Immunoway,YP0141, 1:500
Phospho‐SAPK/**JNK**(Thr183/Tyr185) Rabbit mAb, CST, #4668s, 1:500
Rabbit anti‐**IRS‐1** polyclonal antibody, ABclonal Technology, A16902, 1:500
**IRS‐1**(phosphor Ser307) polyclonal Antibody, Immunoway, YP0146, 1:500
Phospho‐**NF‐κB** p65 (Ser536) (93H1) Rabbit mAb, BestBio, #3033, 1:500
AKT Rabbit mAb, Cell Signaling Technology(CST), #4691s, 1:1000
Phospho‐**AKT**(Ser473) Rabbit mAb, Cell Signaling Technology(CST), #4046s, 1:500
HRP‐linked goat anti‐rat antibody, Antgene, 1:2000
HRP‐linked goat anti‐rabbit antibody, Antgene, 1:2000
HRP‐linked goat anti‐mouse antibody, Antgene, 1:2000
HRP‐linked rabbit anti‐goat antibody, Antgene, 1:2000
Commercial kits
CBA BD Cytometric Bead Array (CBA) Mouse **Inflammation Kit**. Becton, Dickinson and Company(BD), 552 364
Mouse **Insulin** ELISA Kit, Bio‐Swamp, MU30432
**Hb kit**, Nanjing Jiancheng Bioengineering Institute, A056
**Nuclear Protein Extraction** Kit/BestBio BB‐3102‐1

Abbreviations: Ano1, anoctamin 1; HRP, horseradish peroxidase; IL, interferon; SCF, stem cell factor; TLR4, toll‐like receptor 4; TNF, tumor necrosis factor

The nuclear protein extraction kit (Nuclear Protein Extraction Kit/BestBio [BB‐3102‐1]) was used in nuclear protein extraction.

### 2.11 Real‐time reverse transcription polymerase chain reaction

The RNA of tissues (20 mg of colon tissues [a random segment of colon], 20 mg of liver tissues, and 20 mg of muscle tissues) was isolated using 700ul of TRIzol reagent (Takara, Otsu, Japan,) and then reverse transcribed to cDNA by Prime Script™ RT Master Mix (Perfect Real‐Time; Takara, Otsu, Japan). Quantitative polymerase chain reaction (PCR) amplification was performed with a 10 μL final reaction mixture consisting of 1 μl cDNA, 5 μl SYBR‐Green reaction mix (Takara, Otsu, Japan), 0.5 μl of each sense and antisense primer (both from Invitrogen), and 3 μl of sterile water performed by the Roche LightCycler R480 (Roche, Switzerland). The PCR conditions were initial denaturation at 95°C for 5 min and followed by 45 cycles of PCR reaction: denaturation at 95°C for 20 s, annealing at 58°C for 30 s and elongation at 72°C for 30 s. Gene expression was normalized by GAPDH level and relative changes in target gene expression were determined using the 2^−ΔCt^ method (ΔCt = CT target gene ‐ CT GAPDH). The Primer sequences as shown in Table 2.

**TABLE 2:** Primer sequences

Primer sequences		
**mSCF**	Forward	5′‐GGAAAATAGTGGATGACCTCGTG‐3′
	Reverse	5′‐TGGAATCTTTCTCGGGACCTAAT‐3′
**c‐Kit**	Forward	5′‐CAGAGGCTTAGCGGAGTGAA‐3′
	Reverse	5′‐AGGGCAAGGACAAGGGAAC‐3′
**ZO‐1**	Forward	5′‐AGGACACCAAAGCATGTGAG‐3′
	Reverse	5′‐GGCATTCCTGCTGGTTACA‐3′
**Occludin**	Forward	5′‐ACGGACCCTGACCACTATGA‐3′
	Reverse	5′‐TCAGCAGCAGCCATGTACTC‐3′
**Claudin‐1**	Forward	5′‐GCTGGGTTTCATCCTGGCTTCT‐3′
	Reverse	5′‐CCTGAGCGGTCACGATGTTGTC‐3′
**IL‐1β**	Forward	5′‐AAGGGCTGCTTCCAAACCTTTGAC‐3′
	Reverse	5′‐TGCCTGAAGCTCTTGTTGATGTGC‐3′
**IL‐6**	Forward	5′‐TCCTACCCCAATTTCCAATGCT‐3′
	Reverse	5′‐TGAATTGGATGGTCTTGGTCCTT‐3′
**IL‐10**	Forward	5′‐TGCTATGCTGCCTGCTCTTA‐3′
	**Reverse**	**5′‐TCATTTCCGATAAGGCTTGG‐3′**
**TNF‐α**	Forward	5′‐AGGGTCTGGGCCATAGAACT‐3′
	Reverse	5′‐CCACCACGCTCTTCTGTCTAC‐3′
**TLR4**	Forward	5′‐AGAAATTCCTGCAGTGGGTCA‐3′
	Reverse	5′‐TCTCTACAGGTGTTGCACATGTCA‐3′
**GAPDH**	Forward	5′‐AGGTCGGTGTGAACGGATTTG‐3′
	Reverse	5′‐TGTAGACCATGTAGTTGAGGTCA‐3′

Abbreviations: IL, interferon; mSCF, membrane‐bound stem cell factor; TLR4, toll‐like receptor 4; TNF, tumor necrosis factor; ZO‐1, zonula occludens‐1

### 2.12 Fecal microbiota transplantation and randomization

C57BL/6 male mice were treated with a cocktail of antibiotics (ABX; metronidazole 1 g/L, vancomycin 500 mg/L, neomycin 1 g/L, ampicillin 1 g/L in drinking water) to deplete their gut microflora every day for 4 weeks and then were induced successfully to the T2D mice model (high‐fat feeding for 4 weeks and then intraperitoneal injection of STZ at the dose of 100 mg/Kg), which could be used as recipients of fecal microbiota transplantation (FMT). For transplantation, fecal matter from donor mice (previous experimental groups) was administered by oral gavage twice per week for 4 weeks to the recipient mice.^15^ Briefly, one fecal pellet from each donor was collected, dissolved in sterile PBS (2 ml), centrifuged at 500 g for 30″, and then the suspension was administered by oral gavage (200 μl/mouse). The groups were showed in Figure 1, program 2.

### 2.13 Kit^W/Wv^ DM mice model and EA treatment

Sixteen male Kit^W/Wv^ mice received high‐fat diet for 4 weeks followed by an intraperitoneal injection of STZ (100 mg/kg) to induce the T2D model. Those mice were randomly divided into five groups: normal control group, Kit^W/Wv^ group, Kit^W/Wv^ + DM group, Kit^W/Wv^ + DM + SEA group, and Kit^W/Wv^ + DM + EA group. The blood glucose level was monitored once every 2 weeks. Eight weeks after the EA treatment, the total gastrointestinal transit and fecal parameter were detected, and the c‐Kit and mSCF expression level in the colon were assessed. In addition, the gut microbiota was detected.

### 2.14 Statistical analysis

Multiple comparisons were analyzed by one‐way analysis of variance followed by Tukey–Kramer test. Bioinformatic analysis of the gut microbiota was carried out using the Majorbio Cloud platform. Incremental AUC was calculated for glucose and insulin. A value *p* < .05 could be regarded as statistically significant. All experiments were repeated more than two times and the results were showed as mean ± SEM. We used SPSS 17.0 (SPSS Inc. Chicago, IL) to calculate the statistics.

## 3 RESULTS

### 3.1 The EA improved the glycemic profile of diabetic mice, and the FMT influenced glycemic profile

**FIGURE 2:**
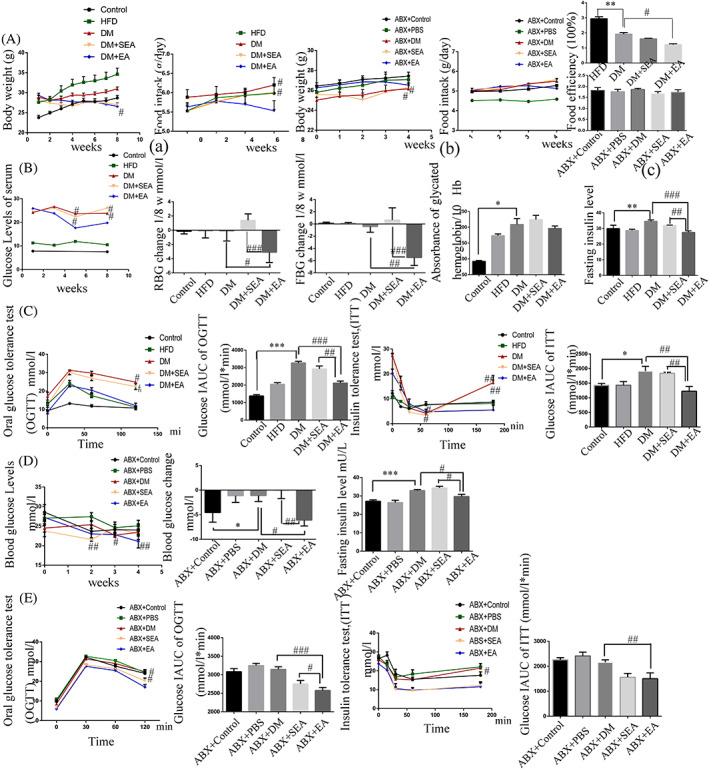
The effects of EA and FMT on glycemic profile. (A). Body weight, food intake (a, b), and food efficiency (c) with EA treatment and FMT. B, C. The effects of EA on glycemic profile: (B). Assessment of the blood glucose among groups once every 2 weeks; the total reduction of blood glucose level (RBG and FBG) after the EA intervention; and the levels of serum HbA1c and fasting insulin. (C). OGTT, ITT, and calculation of the AUC. *N* = 6 in each group. D, E. The effects of the FMT on glycemic profile: (D). Assessment of the blood glucose status weekly, the total reduction of blood glucose level, and the level of serum fasting insulin. (E). OGTT, ITT, and calculation of the AUC. *N* = 6 in each group. *p < .05, **p < .01, ***p < .001, compared with control group. #*p* < .05, ##*p* < .01, ###*p* < .001 compared with the DM group and the DM + SEA group. ABX, antibiotics; AUC, area under the curve; DM, diabetic mice; EA, electroacupuncture; FBG, fasting blood glucose; FMT, fecal microbiota transplantation; HbA1c, glycosylated hemoglobin; HFD, high‐fat diet; IAUC, incremental area under the curve; ITT, insulin tolerance test; OGTT, oral glucose tolerance test; RBG, random blood glucose; SEA, sham electroacupuncture

As shown in Figure 2A, body weight, food intake and food efficiency were slightly reduced in the DM + EA group (*p* = .34, *p* = .48, *p* = .35). As shown in Figure 2B, the mice in the EA group had a significant reduction in RBG level (*p* = .019) and FBG level (*p* = .005) compared with mice in the DM group. The glycosylated hemoglobin level of mice with EA treatment did not show significant difference from that in the DM group. The serum insulin level of EA group was greatly reduced (*p* < .001, compared with the DM group).

OGTT and ITT are other measures to show the insulin sensitivity related to glucose homeostasis (Figure 2C). The blood glucose level in OGTT fell at the faster rate in the EA treated mice than the DM group at 60 min and 120 min (*p* = .002, *p* = .026, respectively). As for ITT, the EA group showed a slower increase of blood glucose after 60 min, especially at 60 min and 180 min compared to the DM group (*p* = .049, *p* = .002, resp.). AUCs of OGTT and ITT in the DM group were higher than the control group (*p* < .001, *p* = .023, respectively), but they were lower than the EA group (*p* < .001, *p* = .001, respectively).

Body weight was slightly reduced, food intake and food efficiency were not changed in the ABX + EA group compared to the ABX + DM group (Figure 2). As shown in Figure 2D, the recipient mice that received the fecal microbiota from the EA‐treated mice showed a declining blood glucose level compared with the ABX + DM group (*p* = .021). The level of serum insulin of mice in the ABX + EA group were lower than the ABX + DM group (*p* = .029). As for OGTT (Figure 2D), the blood glucose level of mice that received microbiota from the EA‐treated mice fell at a faster rate in than in the ABX + DM group (*p* = .049) at 120 min. According to the results of ITT (Figure 2E), we found that the blood glucose level had a slower increase in the ABX + EA groups than the ABX + DM group after 60 min especially at 120 min (*p* = .026). AUC of OGTT and ITT in the ABX + EA group were lesser compared with the ABX + DM group (*p* < .001, *p* = .006, respectively).

### 3.2 The EA altered the intestinal flora of diabetic mice

**FIGURE 3:**
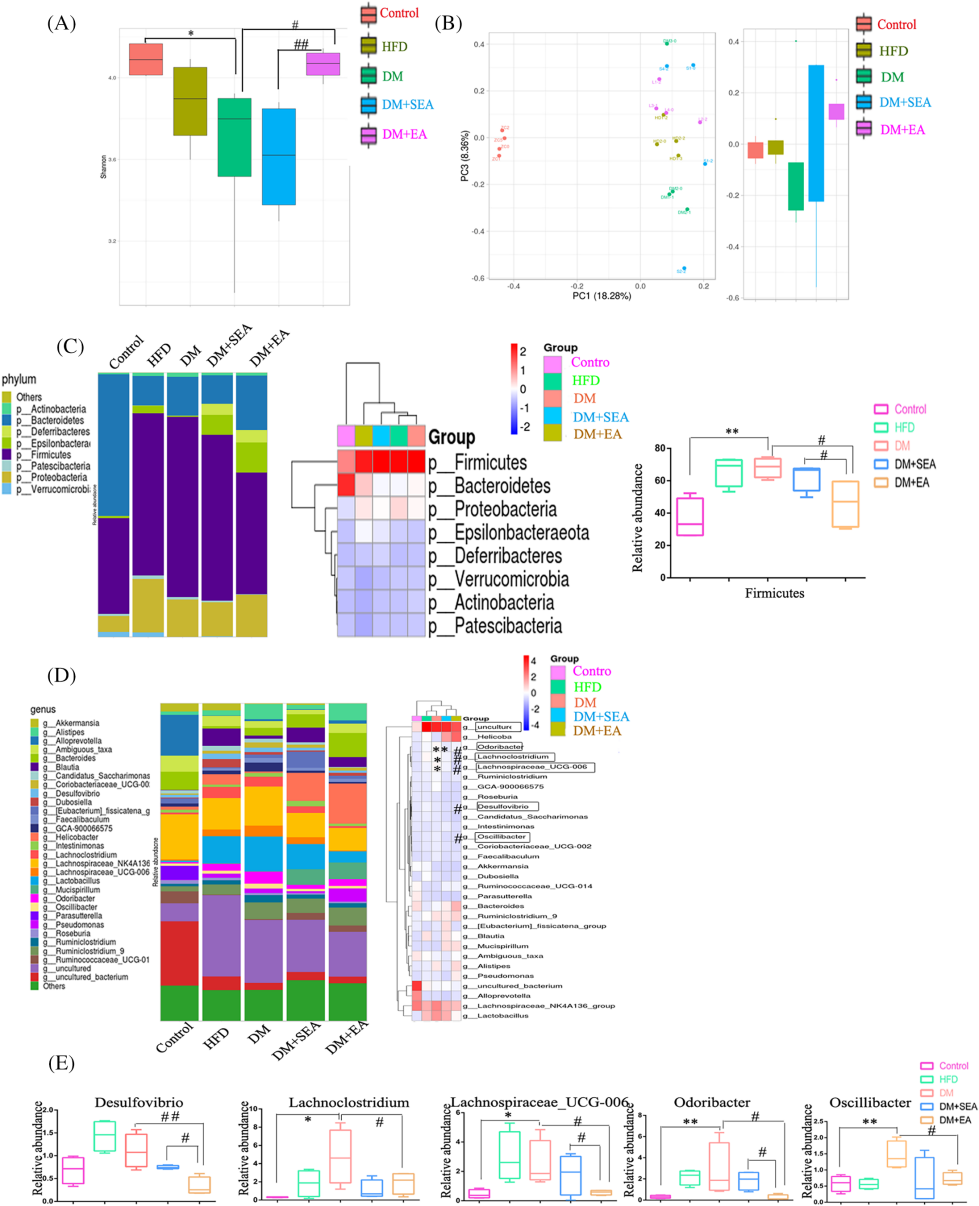
The changes of gut microbiota. (A). Alpha diversity: Shannon‐Wiener diversity index. (B). PCoA analysis (PC3/PC1). (C). Phylum abundance, heatmap, and major bacterial phyla Firmicutes proportion. (D). Species abundance and species heatmap at the genus level. (E). The change of some gut microflora at the genus level. *N* = 6 in each group. *p < .05, **p < .01, ***p < .001, compared with control group. #*p* < 0.05, ##*p* < 0.01, ###*p* < 0.001 compared with the DM group and the DM + SEA group. DM, diabetic mice; EA, electroacupuncture; HFD, high‐fat diet; PCoA, principal coordinate analysis; SEA, sham electroacupuncture

As shown in Figure 3A, Shannon‐Wiener index decreased a lot in the DM group (*p* = .025), and it increased noticeably after the EA treatment (*p* = .034, compared with the DM group). Principal coordinate analysis (PCoA) represented that gut microbiota structure of the EA‐treated group showed a significant deviation from that of the DM group but approached that of the control group (Figure 3B.). At the Phyla level (Figure 3C), there was an extended abundance of Firmicutes in the DM group compared with the control group (*p* = .001), but the EA treatment reduced it (*p* = .012, compared with the DM group).

At the genus level (Figure 3D,E), the microbiota of diabetic mice was comparatively increased in *Lachnoclostridium*, *Lachnospiraceae_UCG‐006*, *Odoribacter*, and *Oscillibacter* (*p* = .034, *p* = .034, *p* = .002, *p* = .008, resp. compared with the control group), all of which were reduced in the EA‐treatment mice (*p* = .045, *p* = .045, *p* = .030, *p* = .021, respectively compared with the DM group). The EA decreased *Desulfovibrio* of the mice compared with the DM group (*p* = .001).

### 3.3 The EA enhanced the intestinal epithelial barrier and balanced the inflammatory cytokines in the colon and serum

**FIGURE 4:**
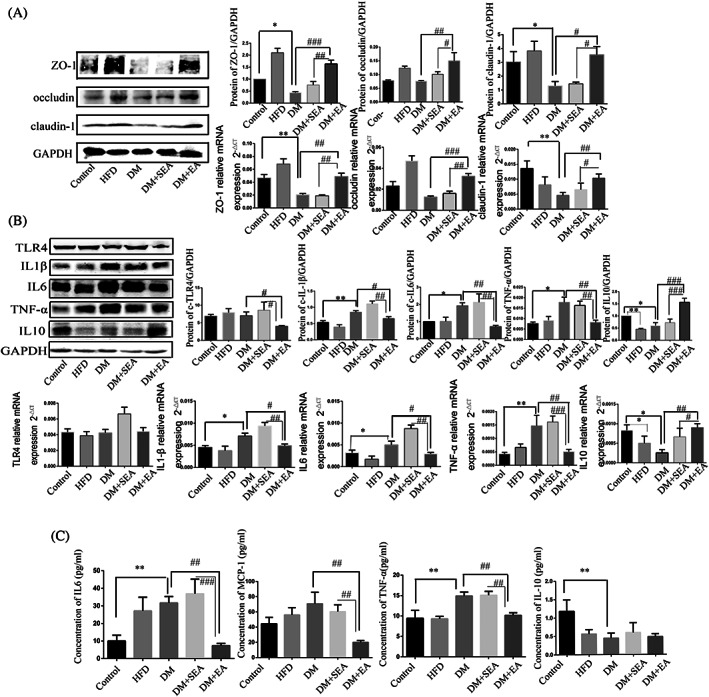
The effects of EA on the intestinal epithelial barrier and the inflammatory cytokines. (A). The tight junction protein expression level and the mRNA level of such as ZO‐1, occludin, and claudin‐1 in the colon. (B). The protein and mRNA level of TLR4, IL‐1β, IL‐6, IL‐10, and TNF‐α in the colon. (C). Inflammatory cytokines level in serum such as IL‐6, MCP‐1, TNFα, and IL‐10. *N* = 6 in each group. *p < .05, **p < .01, ***p < .001, compared with control group. #*p* < .05, ##*p* < .01, ###*p* < .001 compared with the DM group and the DM + SEA group DM, diabetic mice; EA, electroacupuncture; HFD, high‐fat diet; IL, interleukin; MCP‐1, monocyte chemoattractant protein‐1; SEA, sham electroacupuncture; TLR4, toll‐like receptor 4; TNF, tumor necrosis factor; ZO‐1, zonula occludens‐1

The proteins of zonula occludens‐1 (ZO‐1; *p* = .011) and claudin‐1 (*p* = .049) were seriously reduced in the DM group compared with that in the control group, but they were upregulated in the EA group compared with the DM group (*p* < .001, *p* = .016). There was no obvious change of occludin protein level between the control group and DM group, but the EA treatment could induce the higher expression of occludin protein than the DM group (*p* = .002). Meanwhile, the relative mRNA expression of ZO‐1 (*p* = .003), occludin (*p* < .001) and claudin‐1 (*p* = .007) in the colons of EA‐treated mice was higher than the DM group (Figure 4A).

As shown in Figure 4B, toll‐like receptor 4 protein level was downregulated in the EA group compared with the DM group (*p* = .03). In the EA group, the protein levels of IL‐1β (*p* = .045), IL‐6 (*p* = .005), and TNF‐α (*p* = .003) were dramatically reduced compared with the DM group. However, the expression level of IL‐10 in the mice of the EA group was higher thanin the DM group (*p* < .001). The changes of mRNA expression of IL‐1β (*p* = .036), IL‐6 (*p* = .048), TNF‐α (*p* = .004), and IL‐10 (*p* = .007) were similar to that of protein in the EA group, compared with the DM group.

As shown in Figure 4C, the level of IL‐6 (*p* = .001) and TNFα (*p* = .002) in the serum was higher in the DM group than the controls. The EA decreased the levels of IL‐6 (*p* = .005), MCP‐1 (*p* = .001), and TNFα (*p* = .008) compared with the DM group. The serum level of IL10 was decreased in the DM group compared with the control group (*p* = .001), but the EA treatment had no influence on it.

### 3.4 The EA regulated the IKKβ/NF‐κB‐JNK‐IRS‐1‐AKT signaling in the liver and skeletal muscles

**FIGURE 5:**
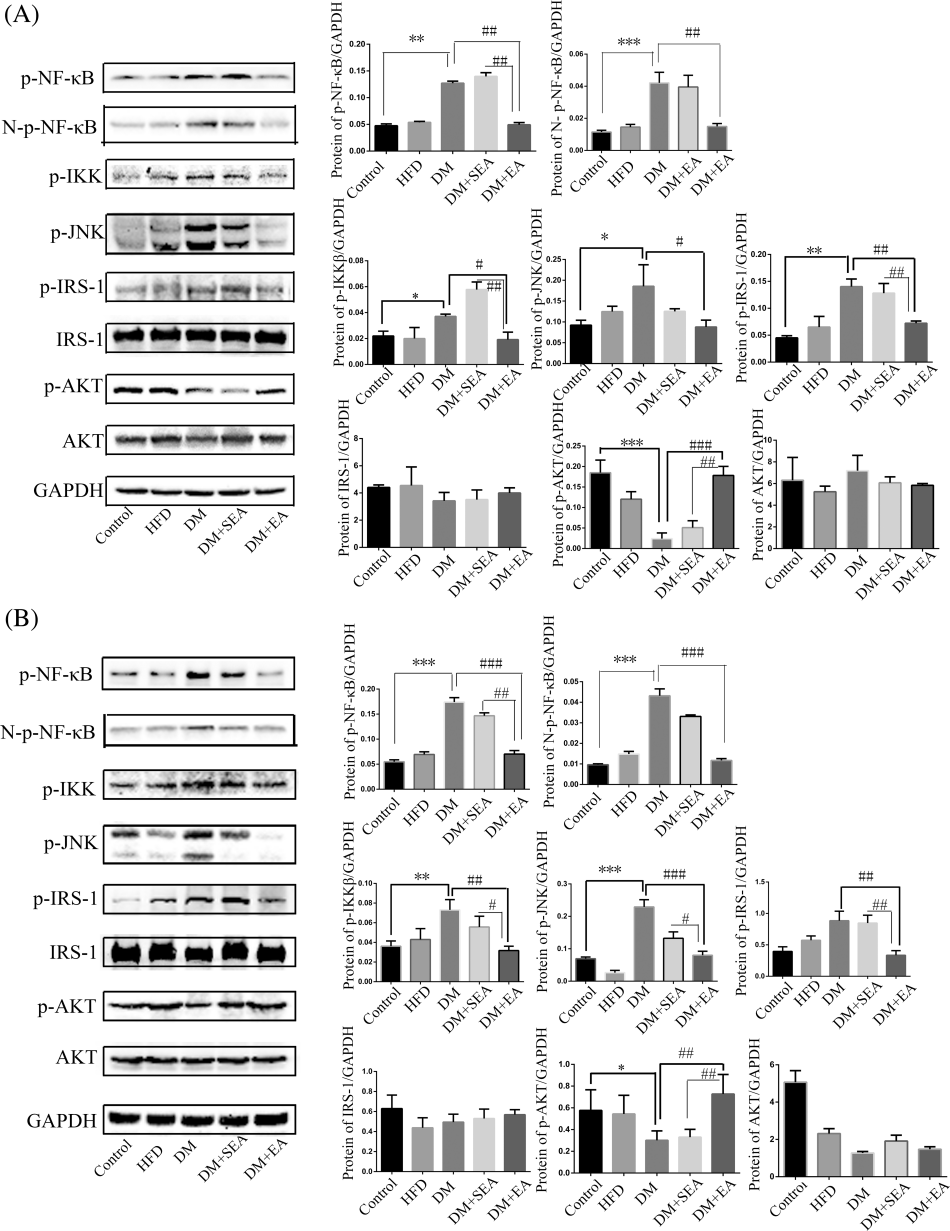
The effects of EA on the IKKβ/NF‐κB‐JNK‐IRS‐1‐AKT signaling. (A). The protein levels of total p‐NF‐κB, Nuclear‐P‐NF‐κB，p‐IKKβ, p‐JNK, p‐IRS‐1, total IRS‐1, p‐AKT, and total AKT. *N* = 6 in each group. (B). The effects of EA on the IKKβ/NF‐κB‐JNK‐IRS‐1‐AKT signaling in the skeletal muscle. The EA inhibited the phosphorylation of IKKβ, total NF‐κB, Nuclear NF‐κB, JNK, and IRS‐1. The EA increased the phosphorylation of AKT protein. *N* = 6 in each group. *p < .05, **p < .01, ***p < .001, compared with the control group. #*p* < .05, ##*p* < .01, ###*p* < .001 compared with the DM group and the DM + SEA group. DM, diabetic mice; EA, electroacupuncture; HFD, high‐fat diet; SEA, sham electroacupuncture

The protein expression levels of total p‐NF‐κB, Nuclear‐p‐NF‐κB (N‐p‐NF‐κB), p‐IKKβ, p‐JNK, and p‐IRS‐1 were increased in the DM group (*p* = .008, *p* < .001, *p* = .044, *p* = .028, *p* < .001, respectively compared with the control group), but downregulated with the EA treatment (*p* = .006, *p* = .004, *p* = .036, *p* = .022, *p* = .002, respectively compared with the DM group). What is more, EA enhanced the expression level of p‐AKT (*p* < .001, compared with the DM group) as shown in Figure 5A.

In the skeletal muscles (Figure 5B), the EA effectively reduced the phosphorylation level of total p‐NF‐κB, Nuclear‐p‐NF‐κB, IKKβ, JNK, and IRS‐1 (*p* < .001, *p* < .001, *p* = .004, *p* < .001, *p* = .001, respectively compared with the DM group). In contrast, the EA treatment enhanced the phosphorylation level of AKT (*p* = .006, compared with the DM group).

### 3.5 The EA promoted the gastrointestinal motility and mSCF/c‐Kit expression of diabetic mice, instead of in the kit^W/Wv^ DM mice

**FIGURE 6:**
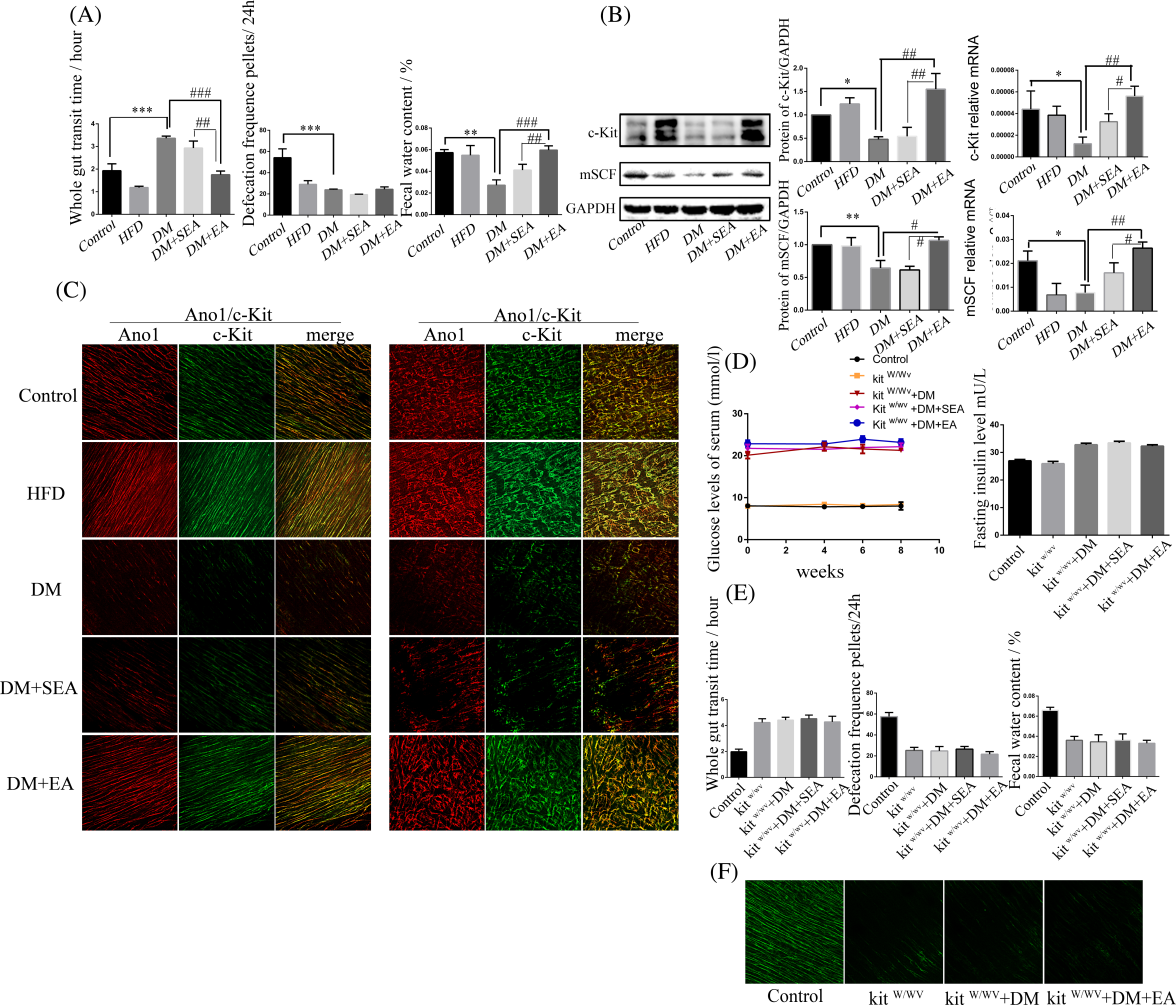
The effects of EA on the GI motility and mSCF/c‐Kit expression. A‐C. In the diabetic mice model: (A). Evaluation of the GI motility: the whole gut transit time, defecation frequencies pellets for 24 h, and fecal water content. (B). The protein and mRNA levels of mSCF and c‐Kit in the colon. *N* = 6 in each group. *p < .05, **p < .01, ***p < .001, compared with the control group. #*p* < .05, ##*p* < .01, ###*p* < .001 compared with the DM group and the DM + SEA group. (C). The immunofluorescence intensity of Ano1 and c‐Kit in the colon with the EA treatment. *N* = 6 in each group. D‐F. In the kit^W/Wv^ DM mice: (D). The effects of EA on the blood glucose level and insulin. (E). The effects of EA on the gut transit, defecation frequency and fecal water content. (F). The effect of EA on ICC expression. *N* = 4 in each group. Ano1, anoctamin 1; DM, diabetic mice; EA, electroacupuncture; GI, gastrointestinal; HFD, high‐fat diet; ICC, interstitial cells of Cajal; mSCF, membrane‐bound stem cell factor; SEA, sham electroacupuncture

As shown in Figure 6A, the mice in the DM group had delayed gut transit (*p* < .001), reduced defecation frequency (*p* < .001), and decreased fecal water content (*p* = .001), compared with the control group. Furthermore, the EA could accelerate gut transit (*p* < .001) and fecal water content (*p* < .001), compared with the DM group.

The expression levels of c‐Kit and mSCF in the colon were shown in Figure 6B. The protein and mRNA expression levels of c‐Kit in the DM group were reduced compared with the control group (*p* = .043, *p* = .036, respectively). Nevertheless, the EA could improve the c‐Kit protein (*p* = .002) and mRNA level (*p* = .005) compared with the DM group. The expression of mSCF had the similar trend with the c‐Kit.

c‐Kit immunoreactivity intensity of colon (Figure 6C, ICC‐IM and ICC‐MY) in the DM group was lower than in the control group but increased in the EA group, compared with the DM group.

As shown in Figure 6D, the EA could not significantly reduce the blood glucose and insulin level of the kit^W/Wv^ DM mice. The gut transit, defecation frequency, and fecal water content were not changed by the EA treatment, compared with the kit^W/Wv^ + DM group (Figure 6E). The EA could not increase the expression of c‐kit, compared with the kit^W/Wv^ + DM group (Figure 6F).

### 3.6 The EA did not alter the intestinal flora in the kit^W/Wv^ DM mice

**FIGURE 7:**
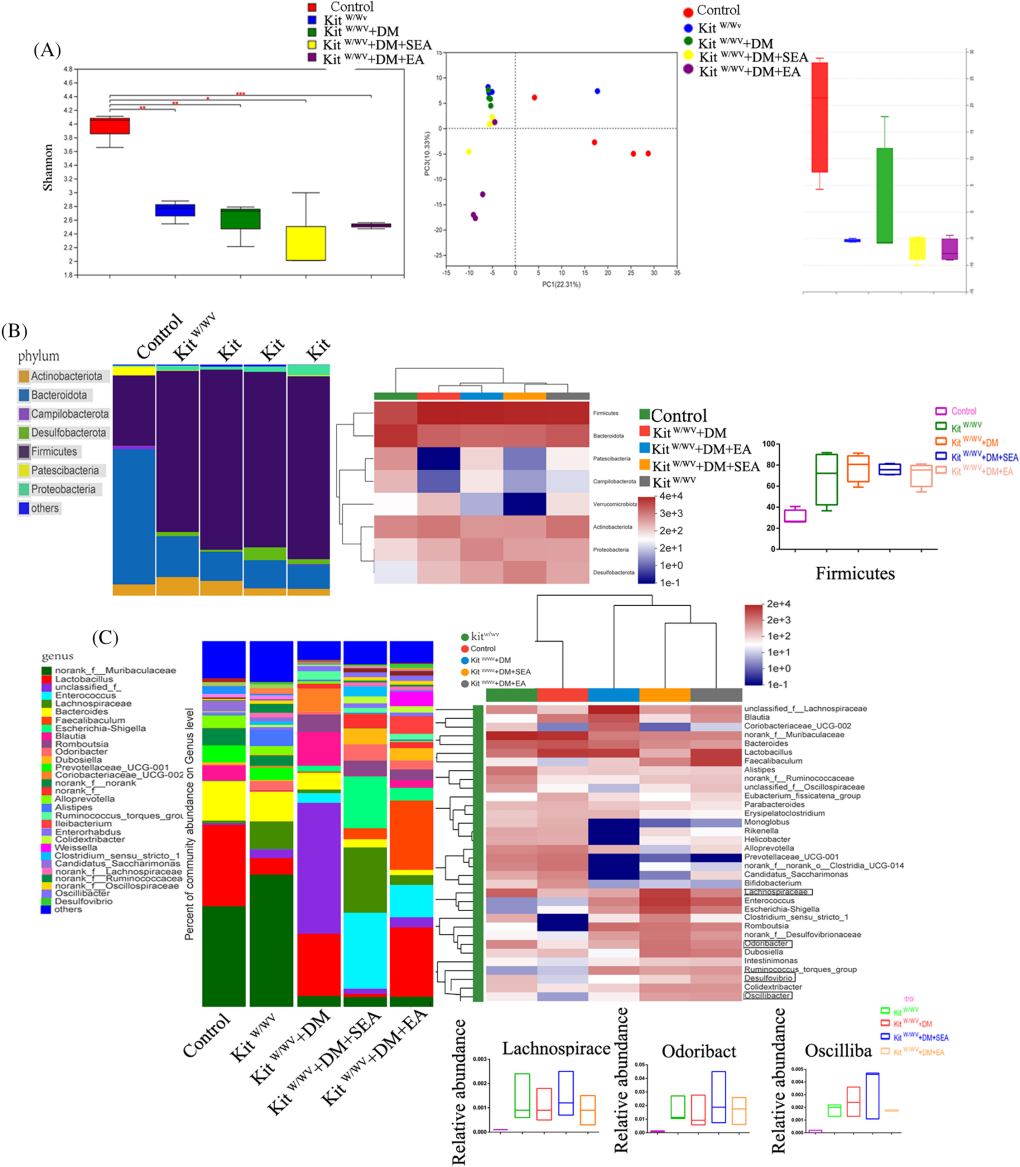
The changes of gut microbiota in the kit‐deficient mice. (A). Shannon‐Wiener diversity index and PCoA (PC3/PC1). (B). Phylum abundance, heatmap and major bacterial phyla Firmicutes proportion. *N* = 4 in each group. ***p < .001, compared with the control group. (C). Species abundance, Species heatmap and some gut microflora at the genus level in the kit‐deficient mice. *N* = 4 in each group. ***p < .001, compared with the control group. DM, diabetic mice; EA, electroacupuncture; PCoA, principal coordinate analysis; SEA, sham electroacupuncture

The EA did not increase the Shannon‐Wiener index and the abundance of Firmicutes in the kit‐deficient mice with DM, and the gut microbiota structure of the EA‐treated group showed no difference from the kit^W/Wv^ + DM group (Figure 7A,B). At the genus level (Figure 7C), the microbiota of mice in the kit^W/Wv^ + DM and kit^W/Wv^ groups were comparatively increased in *Lachnospiraceae_UCG‐006*, *Desulfovibrio*, *Odoribacter*, and *Oscillibacter* (all *p* < .001 compared with the control group), but all of which were not decreased in the EA‐treatment mice (*p* > .05 compared with the kit^W/Wv^ + DM group). *Lachnoclostridium* in mice of kit^W/Wv^ + DM group had a great individual difference so that it was difficult to quantify.

## 4 DISCUSSION

In this study, we first proved the hypoglycemic effect of the EA through regulation of the intestinal microbiota in the T2D mice, as well as its specific mechanism. Then the ICC‐deficient mice model was used to explore the mechanism of the EA on the intestinal flora. Our study demonstrated the EA maintained ICC to promote intestinal motility, thereby regulating intestinal flora to improve glycometabolism inT2D mice.

Individuals with T2D quadrupled in the last 30 years, and T2D has become the ninth leading cause of death.^16^ In contrast to drugs, EA is a potential therapy for the diseases related to insulin resistance for its low cost, relative safety, and effectiveness.^17^ Copious literature indicated that low‐frequency EA (2‐15HZ) had a better effect on metabolism syndrome,^18^, ^19^ and our preliminary experiment found that the EA with high frequency (100HZ) was not effective on blood glucose control in the mice with T2D. Therefore, in this study, we applied low‐frequency (10 HZ) EA at ST36 in the T2D mice to explore its therapeutic effect on blood glucose control. In our study, we also revealed EA at ST36 decreased the blood glucose level of T2D mice, and the hypoglycemic effect of EA was dependent on the reduction of insulin resistance.

However, the mechanisms of the hypoglycemic effect of the EA are unclear, which need to be explored in our follow‐up study. In recent years, increasing research reveals that the pathogenesis of T2D is associated with the intestinal flora.^20^, ^21^ The abundance ratio of intestinal Firmicutes to Bacteroides (F/B) in the patients with insulin resistance was elevated, with greater abundance of opportunistic pathogens, such as *Odoribacter*, *Lachnospiraceae*, and *Oscillibacter*.^22^, ^23^ Addition of probiotics to the diet of diabetic patients could reduce their FBG and HbA1c levels.^24^ Interestingly, a small amount of studies reported that the EA could change the composition of intestinal microbiota, increasing *Lactobacillus* and *Bifidobacterium*.^25^ Similarly, in our study, the EA could increase the flora diversity of diabetic mice, and the EA restored the microbial community structure in diabetic mice. What is more, the EA decreased the proportion of Firmicutes and some specific bacteria, such as *Lachnoclostridium*, *Lachnospiraceae_UCG‐006*, *Odoribacter*, *Oscillibacter*, and *Desulfovibrio*, which were regarded to be susceptible to the T2D. We applied the FMT to further explore whether EA improved glycometabolism through the regulation of gut microbiota. The mice that received microbiota from the EA group showed a better blood glucose control and higher insulin sensitivity, which suggested that the EA may improve the glycometabolism by regulating the intestinal flora. However, the mice that received microbiota from the control group, PBS, and DM group had no significant improvement in blood sugar and insulin sensitivity. We speculate that PBS had no microbiota and DM lacked some beneficial bacteria, whereas EA jas changed some other components such as short chain fatty acids, to be superior to normal fecal bacteria in improving blood glucose and insulin levels, which could also be part of our ongoing research.

Nevertheless, how did the intestinal flora changed by the EA regulate glucose metabolism? Studies found that beneficial bacteria can promote the intestinal barrier function by enhance the tight connection of the epithelium, which balanced the inflammatory state in the intestinal mucosa.^26^, ^27^ Individuals with insulin resistance manifested systematic chronic inflammation as a result of destroyed intestinal barrier function.^28^ Our previous studies have proved that EA could enhance the intestinal barrier and relieve the inflammatory response in mice with dextran sulfate sodium‐induced colitis.^12^ In this study we demonstrated that EA could strengthen the intestinal epithelial barrier by increasing the expression of ZO‐1, occluding, and claudin‐1. What is more, EA could reduce the produce of proinflammatory cytokines and increase the anti‐inflammatory cytokines, suggesting a reduced inflammatory state with EA treatment.

It is well known that alleviating systemic inflammation can promote normal transmission of insulin to reduce the glucose level. Studies have reported that inflammatory factors could induce the insulin resistance via the IKKβ/NF‐κB‐JNK‐IRS‐1‐AKT signal pathway in the liver and muscles. Inflammatory cytokines can activate phosphorylation of IKKβ/NF‐κB‐JNK‐IRS‐1 pathway^29^, ^30^ and then suppress phosphorylation of AKT, resulting in the reduced glucose uptake into cells, ultimately weakening insulin sensitivity.^31^ There are no reports on the role of EA in regulating the IKKβ/NF‐κB‐JNK‐IRS‐1‐AKT pathway. Our results first verified EA could promote insulin sensitivity by regulating IKKβ/NF‐κB‐JNK‐IRS‐1‐AKT signaling in the liver and muscles. Above all, we considered EA could enhance the intestinal barrier function by regulating the intestinal flora, then reduce the inflammatory state of the system, ultimately lower blood glucose level through regulation of IKKβ/NF‐κB‐JNK‐IRS‐1‐AKT signaling.

However, the specific mechanism of EA to regulate the intestinal flora needs further investigation. Some profiles had elaborated the close relationship between gastrointestinal (GI) motility and gut microbiota. In the zebrafish model of intestinal dysmotility induced by intestinal neuron gene mutations, the proportion between two strains of intestinal flora (*Aeromonas* vs *Vibrio*) had reversed.^32^ Cisapride inhibited excessive proliferation of Gram‐negative bacteria by promoting intestinal motility in a cirrhotic model.^33^ Our previous studies have confirmed that diabetic mice exhibited insufficient ICC and intestinal dysmotility, and EA treatment could improve GI function by increasing the ICC.^14^, ^34^ We assumed that EA regulated gut microbiota in T2D mice probably by promoting GI movement via ICC repair. In our results, EA could increase colonic ICC expression and improve intestinal motility in T2D mice. Kit^W/Wv^ mice in our study exhibited the deficiency in colonic ICC and disorder in gut motility. We discovered the EA treatment could not alter the blood glucose level, diversity, and composition of microflora in Kit^W/Wv^ DM mice, which indicated that the effect of EA on gut microbiota may be mediated by ICC‐dependent GI motility.

We concluded that the EA could increase intestinal motility by increasing ICC expression, thereby regulating the intestinal flora, enhancing the intestinal epithelial barrier function, and reducing the level of systemic inflammation, and ultimately regulating the IKKβ/NF‐κB‐JNK‐IRS‐1‐AKT signal pathway in the liver and muscles to improve glucose metabolism.

## FUNDING INFORMATION

The National Natural Science Foundation of China (nos.81570488; nos. 8177031019).

## DISCLOSURE

The authors report no conflicts of interest in this work. The institutional ethics committees have approved this research comply with acceptable international standards (LACUC Number: 826).

## Data Availability

The data used to support the findings of this study are available from the corresponding author upon request.

## References

[1] Ishizaki N , Okushi N , Yano T , Yamamura Y . Improvement in glucose tolerance as a result of enhanced insulin sensitivity during electroacupuncture in spontaneously diabetic Goto‐Kakizaki rats. Metabolism. 2009;58(10):1372‐1378. 19501858 10.1016/j.metabol.2009.05.001

[2] Liang F , Chen R , Nakagawa A , et al. Low‐frequency Electroacupuncture improves insulin sensitivity in obese diabetic mice through activation of SIRT1/PGC‐1alpha in skeletal muscle. Evid Based Complement Alternat Med. 2011;2011:735297. 20981161 10.1155/2011/735297 PMC2964507

[3] Qin J , Li Y , Cai Z , et al. A metagenome‐wide association study of gut microbiota in type 2 diabetes. Nature. 2012;490(7418):55‐60. 23023125 10.1038/nature11450

[4] Cotillard A , Kennedy SP , Kong LC , et al. Dietary intervention impact on gut microbial gene richness. Nature. 2013;500(7464):585‐588. 23985875 10.1038/nature12480

[5] Wang H , Wang Q , Liang C , et al. Acupuncture regulating gut microbiota in abdominal obese rats induced by high‐fat diet. Evid Based Complement Alternat Med. 2019;2019:4958294. 31275411 10.1155/2019/4958294 PMC6582896

[6] Si YC , Miao WN , He JY , Chen L , Wang YL , Ding WJ . Regulating gut Flora Dysbiosis in obese mice by Electroacupuncture. Am J Chin Med. 2018;1‐17. (Epub ahead of print). 10.1142/S0192415X18500763 30284469

[7] Liu T , Wu Y , Wang L , et al. A more robust gut microbiota in calorie‐restricted mice is associated with attenuated intestinal injury caused by the chemotherapy drug cyclophosphamide. mBio. 2019;10(2):e02903‐e02918. 30862756 10.1128/mBio.02903-18 PMC6414708

[8] Cani PD , Possemiers S , van de Wiele T , et al. Changes in gut microbiota control inflammation in obese mice through a mechanism involving GLP‐2‐driven improvement of gut permeability. Gut. 2009;58(8):1091‐1103. 19240062 10.1136/gut.2008.165886 PMC2702831

[9] Carvalho BM , Guadagnini D , Tsukumo DML , et al. Modulation of gut microbiota by antibiotics improves insulin signalling in high‐fat fed mice. Diabetologia. 2012;55(10):2823‐2834. 22828956 10.1007/s00125-012-2648-4

[10] Cui X , Qian DW , Jiang S , Shang EX , Zhu ZH , Duan JA . Scutellariae radix and Coptidis Rhizoma improve glucose and lipid metabolism in T2DM rats via regulation of the metabolic profiling and MAPK/PI3K/Akt signaling pathway. Int J Mol Sci. 2018;19(11):3634. 10.3390/ijms19113634 PMC6274950 30453687

[11] Song S , An J , Li Y , Liu S . Electroacupuncture at ST‐36 ameliorates DSS‐induced acute colitis via regulating macrophage polarization induced by suppressing NLRP3/IL‐1beta and promoting Nrf2/HO‐1. Mol Immunol. 2019;106:143‐152. 30610999 10.1016/j.molimm.2018.12.023

[12] Wang L , An J , Liu S , et al. Electroacupuncture preserves intestinal barrier integrity through modulating the gut microbiota in DSS‐induced chronic colitis. Life Sci. 2020;261:118473. 32971101 10.1016/j.lfs.2020.118473

[13] Kashyap PC , Marcobal A , Ursell LK , et al. Complex interactions among diet, gastrointestinal transit, and gut microbiota in humanized mice. Gastroenterology. 2013;144(5):967‐977. 23380084 10.1053/j.gastro.2013.01.047 PMC3890323

[14] Xu J , Chen Y , Liu S , Hou X . Electroacupuncture regulates apoptosis/proliferation of intramuscular interstitial cells of cajal and restores colonic motility in diabetic constipation rats. Evid Based Complement Alternat Med. 2013;2013:584179. 24348706 10.1155/2013/584179 PMC3852313

[15] Cui B, Su D, Li W, et al. Effects of chronic noise exposure on the microbiome‐gut‐brain axis in senescence‐accelerated prone mice: implications for Alzheimer's disease. J Neuroinflammation. 2018;15(1):190. 10.1186/s12974-018-1223-4 PMC6015475 29933742

[16] Formiga F , Camafort M , Carrasco Sánchez FJ . Heart failure and diabetes: the confrontation of two major epidemics of the 21st century. Rev Clin Esp (Barc). 2020;220(2):135‐138. 30878139 10.1016/j.rce.2019.01.009

[17] Shu Q , Chen L , Wu S , et al. Acupuncture targeting SIRT1 in the hypothalamic arcuate nucleus can improve obesity in high‐fat‐diet‐induced rats with insulin resistance via an anorectic effect. Obes Facts. 2020;13(1):40‐57. 31935731 10.1159/000503752 PMC7105640

[18] Chang SL , Lin JG , Chi TC , Liu IM , Cheng JT . An insulin‐dependent hypoglycaemia induced by electroacupuncture at the Zhongwan (CV12) acupoint in diabetic rats. Diabetologia. 1999;42(2):250‐255. 10064107 10.1007/s001250051146

[19] Johansson J , Feng Y , Shao R , Lönn M , Billig H , Stener‐Victorin E . Intense electroacupuncture normalizes insulin sensitivity, increases muscle GLUT4 content, and improves lipid profile in a rat model of polycystic ovary syndrome. Am J Physiol Endocrinol Metab. 2010;299(4):E551‐E559. 20663984 10.1152/ajpendo.00323.2010

[20] Leiva‐Gea IS‐AL , Martín‐Tejedor B , Castellano‐Castillo D , et al. Gut microbiota differs in composition and functionality between children with type 1 diabetes and MODY2 and healthy control subjects: a case‐control study. Diabetes Care. 2018;41(11):2385‐2395. 30224347 10.2337/dc18-0253

[21] Holmes D . Gut microbiota: antidiabetic drug treatment confounds gut dysbiosis associated with type 2 diabetes mellitus. Nat Rev Endocrinol. 2016;12(2):61. 26668122 10.1038/nrendo.2015.222

[22] Garcia‐Mazcorro JF , Pedreschi R , Chew B , Dowd SE , Kawas JR , Noratto G . Dietary supplementation with raspberry extracts modifies the fecal microbiota in obese diabetic db/db mice. J Microbiol Biotechnol. 2018;28(8):1247‐1259. 29943551 10.4014/jmb.1803.03020

[23] Tun HM , Bridgman SL , Chari R , et al. Roles of birth mode and infant gut microbiota in intergenerational transmission of overweight and obesity from mother to offspring. JAMA Pediatr. 2018;172(4):368‐377. 29459942 10.1001/jamapediatrics.2017.5535 PMC5875322

[24] Ejtahed HS , Mohtadi‐Nia J , Homayouni‐Rad A , Niafar M , Asghari‐Jafarabadi M , Mofid V . Probiotic yogurt improves antioxidant status in type 2 diabetic patients. Nutrition. 2012;28(5):539‐543. 22129852 10.1016/j.nut.2011.08.013

[25] Xu Z , Li R , Zhu C , Li M . Effect of acupuncture treatment for weight loss on gut flora in patients with simple obesity. Acupunct Med. 2013;31(1):116‐117. 22961606 10.1136/acupmed-2012-010209

[26] Forgie AJ , Fouhse JM , Willing BP . Diet‐microbe‐host interactions that affect gut mucosal integrity and infection resistance. Front Immunol. 2019;10:1802. 31447837 10.3389/fimmu.2019.01802 PMC6691341

[27] Zhi C , Huang J , Wang J , et al. Connection between gut microbiome and the development of obesity. Eur J Clin Microbiol Infect Dis. 2019;38(11):1987‐1998. 31367997 10.1007/s10096-019-03623-x

[28] Moreira APTT , Ferreira AB , Peluzio Mdo C , et al. Influence of a high‐fat diet on gut microbiota, intestinal permeability and metabolic endotoxaemia. Br J Nutr. 2012;108(5):801‐809. 22717075 10.1017/S0007114512001213

[29] Park SHLZ , Sui Y , Helsley RN , et al. IKKb is essential for adipocyte survival and adaptive adipose remodeling in obesity. Diabetes Res Clin Pract. 2016;65(6):1616‐1629. 10.2337/db15-1156 PMC4878418 26993069

[30] Liu Y , Xu W , Zhai T , You J , Chen Y . Silibinin ameliorates hepatic lipid accumulation and oxidative stress in mice with non‐alcoholic steatohepatitis by regulating CFLAR‐JNK pathway. Acta Pharm Sin B. 2019;9(4):745‐757. 31384535 10.1016/j.apsb.2019.02.006 PMC6664044

[31] Sun H , Liu X , Long SR , et al. Antidiabetic effects of pterostilbene through PI3K/Akt signal pathway in high fat diet and STZ‐induced diabetic rats. Eur J Pharmacol. 2019;859:172526. 31283935 10.1016/j.ejphar.2019.172526

[32] Wiles TJ , Jemielita M , Baker RP , et al. Host gut motility promotes competitive exclusion within a model intestinal microbiota. PLoS Biol. 2016;14(7):e1002517. 27458727 10.1371/journal.pbio.1002517 PMC4961409

[33] Pardo A , Bartoli R , Lorenzo‐Zuniga V , et al. Effect of cisapride on intestinal bacterial overgrowth and bacterial translocation in cirrhosis. Hepatology. 2000;31(4):858‐863. 10733540 10.1053/he.2000.5746

[34] An J , Li Y , Song S , Liu S . Electroacupuncture promotes the gastrointestinal motility of diabetic mice by CNP/NPR‐B‐cGMP and PDE3A‐cGMP signaling. Neurogastroenterol Motil. 2019;31(4):e13539. 30672071 10.1111/nmo.13539

